# TranSeqAnnotator: large-scale analysis of transcriptomic data

**DOI:** 10.1186/1471-2105-13-S17-S24

**Published:** 2012-12-07

**Authors:** Ranjeeta Menon, Gagan Garg, Robin B Gasser, Shoba Ranganathan

**Affiliations:** 1 Department of Chemistry and Biomolecular Sciences and ARC Centre of Excellence, Macquarie University, Sydney, NSW 2109, Australia; 2 Department of Veterinary Sciences, The University of Melbourne, Werribee, VIC 3030, Australia; 3 Department of Biochemistry, Yong Loo Lin School of Medicine, National University of Singapore, Singapore 117597

## Abstract

**Background:**

The transcriptome of an organism can be studied with the analysis of expressed sequence tag (EST) data sets that offers a rapid and cost effective approach with several new and updated bioinformatics approaches and tools for assembly and annotation. The comprehensive analyses comprehend an organism along with the genome and proteome analysis. With the advent of large-scale sequencing projects and generation of sequence data at protein and cDNA levels, automated analysis pipeline is necessary to store, organize and annotate ESTs.

**Results:**

TranSeqAnnotator is a workflow for large-scale analysis of transcriptomic data with the most appropriate bioinformatics tools for data management and analysis. The pipeline automatically cleans, clusters, assembles and generates consensus sequences, conceptually translates these into possible protein products and assigns putative function based on various DNA and protein similarity searches. Excretory/secretory (ES) proteins inferred from ESTs/short reads are also identified. The TranSeqAnnotator accepts FASTA format raw and quality ESTs along with protein and short read sequences and are analysed with user selected programs. After pre-processing and assembly, the dataset is annotated at the nucleotide, protein and ES protein levels.

**Conclusion:**

TranSeqAnnotator has been developed in a Linux cluster, to perform an exhaustive and reliable analysis and provide detailed annotation. TranSeqAnnotator outputs gene ontologies, protein functional identifications in terms of mapping to protein domains and metabolic pathways. The pipeline is applied to annotate large EST datasets to identify several novel and known genes with therapeutic experimental validations and could serve as potential targets for parasite intervention. TransSeqAnnotator is freely available for the scientific community at http://estexplorer.biolinfo.org/TranSeqAnnotator/.

## Background

Expressed sequence tags or ESTs, derived from complementary DNA (cDNA) libraries provide a low-cost transcriptomic alternative to whole genome sequencing as these are short, unedited, randomly selected single-pass sequence reads of approximately 200-800 base pairs (bp) which represent a small region or a part of nucleotide sequence from a transcribed protein coding or non-coding messenger mRNA. They play vital role in gene identification and verification of gene prediction as they represent the expressed region of a genome. The analysis of EST data can facilitate gene discovery, help in gene structure identification, complement genome annotation, establish the viability of alternative transcripts, direct single nucleotide polymorphism (SNP) characterization and facilitate proteomic exploration [1-3]. They were used as the primary source for human gene discovery in early 1990s [4]. Besides ESTs, millions of sequencing reads of 35-250 bp are generated with the advent of "next-generation" sequencing (NGS) which further help in the study of transcriptome data mainly for neglected organisms and also, understanding different isoforms of an organism at different stages of development. Studies using experimental proteomic approach have shown the identification of proteins in ESP with transcriptome assembly [5]. Many challenges are faced in the areas of bioinformatics analysis in data storage and management solution and developing informatics tools for analysis with the focus on sequence quality scoring, alignment, assembly, and data processing with the advent of short read strategy of NGS [6,7]. A comprehensive analysis pipeline is required to store, organize and annotate ESTs with several computational tools for pre-processing, clustering, assembly into contiguous segments known as contigs and annotation to yield biological information. The web resources available were reviewed for large-scale EST dataset at each step including clustering, assembly, consensus generation and tools for DNA, protein and ES annotation [8]. A number of analysis steps and tools confounded computational strategies to organize and analyse transcriptomic dataset [9] which is compounded by the ability of some tools to handle high-throughput EST data. An evaluation revealed that all available platforms terminated prior to downstream functional annotation, including gene ontologies (GOs), motif/pattern analysis and pathway mapping. Hence, the establishment of a comprehensive large-scale transcriptomic analysis pipeline [9] was required to be developed to keep up with the rapidity with which enormous amounts of sequence data are currently being generated. An urgent need for advanced, high-throughput computational analyses of EST and genomic sequence datasets using automated platforms is highlighted. EST data are been applied to study of functional biomolecules [9,10] but, predicting ES proteins, from ESTs have been uncommon. Excretory/Secretory (ES) products are the molecules excreted or secreted by a cell or an organism that can circulate throughout the body of an organism (e.g., in the extracellular space) or are localized to or released from the cell surface, making them readily accessible to drugs and/or the immune system. ES products cover 8 ± 20% of the proteome of an organism [11] and include molecules of varied functionality, including chemokines, digestive enzymes cytokines, hormones, toxins, antibodies, morphogens, extracellular proteinases and antimicrobial peptides. They are known to be involved in vital biological processes, including cell adhesion, cell migration, cell-cell communication, differentiation, proliferation, morphogenesis and immune responses [12]. Biochemical and immunological studies of parasitic helminths were focussed on ES proteins. Worms secrete biologically active mediators which can transform or customize their niche within the host [13-15] to regulate or to elude immune attack or stimulate a particular host response.

Some platforms terminate at the assembly level, providing contigs and singletons [16] (referred to as rESTs) while other platforms exclusively run nucleotide-based programs with limited annotation at the protein level [17-20]. Based on the benchmarking results, a robust transcriptome analysis pipeline (TranSeqAnnotator) is constructed with contig generation from ESTs and short reads, updated pathway analysis, non-classically secreted protein identification and extensive annotation with an option to select specific analysis phases by users (detailed below). Proteins secreted by classical and non-classical pathways are identified by a combination of computational approaches to predict ESPs. The pipeline accepts ESTs, quality values, protein sequences and short reads as input and provides as output, assembled rESTs and their annotations including gene ontologies, secretory proteins, mapping to protein domains, motifs, metabolic pathways and interaction databases. TranSeqAnnotator (TSA) is available as web service and can be downloaded for local installation.

### Implementation

**Figure 1:**
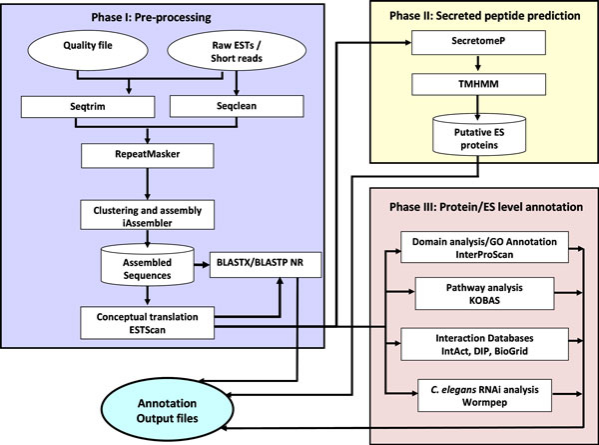
**Schematic diagram of TranSeqAnnotator workflow**.

TranSeqAnnotator workflow has three phases with Phase I (a) for EST or (b) short read fasta sequence pre-processing, assembly, conceptual translation and blast against NR, Phase II for the identification of putative ES proteins, from classically and non-classically secreted proteins and the elimination of transmembrane proteins and Phase III for the combined annotation of the protein sequence and ES proteins involving a carefully selected suite of bioinformatic tools, based on a large-scale transcriptome analysis [21] (Figure 1). TranSeqAnnotator currently implements the genetic codes for 15 organisms, covering the most studied organisms, including human, rat, pig, dog, chicken, rice, wheat, thale cress (*Arabidopsis thaliana*), zebrafish, yeast and a free-living roundworm (*Caenorhabditis elegans*).

Phase I accept ESTs and short reads as well as quality values in the case of ESTs as input for pre-processing and assembly (Figure 1).

The sequence cleaning step uses seqclean [22] and seqtrim [23] with ESTs alone and with ESTs and quality sequences respectively followed by masking the repeats using RepeatMasker [24] which is optional. The Phase I (b) accepts short reads and pre-processing is carried out using seqclean. The masked sequences are then passed on for clustering and assembly with iAssembler http://bioinfo.bti.cornell.edu/tool/iAssembler/ which incorporates MIRA [25] and CAP3 assemblers for ESTs and short reads. For conceptual translation into proteins, the program ESTScan [26] applies the genetic code from the nearest organism to the contig and singleton sequences generated by CAP3 or iAssembler.

In Phase II, the protein sequences generated in Phase I, using TMHMM [27] and putative ES proteins identified using SecretomeP [28] are annotated (Figure 1). Firstly, the signal sequence is checked with SignalP while, SecretomeP looks for non-classically secreted proteins and the hidden Markov model probability scores (SignalPNN and SignalP-HMM), using default parameters that can be modified by experienced users. Subsequently, all proteins with signal sequences are passed on to TMHMM, a hidden Markov model-based transmembrane helix prediction program, to ''filter out'' of transmembrane proteins. ES proteins, the subset lacking transmembrane helices are further annotated. Phase III, the annotation level for protein sequences or ES proteins comprises a suite of computational tools InterProScan [29] for domain analysis and Gene Ontology, pathway mapping using KOBAS (KEGG Orthology-Based Annotation System) [30,31]. Also, protein BLAST is employed to search databases derived from Wormpep [32] for locating nematode homologues and a list of homologous proteins in *C. elegans*, archived in WormBase as well as interaction databases like IntAct [33], BioGrid [34] and DIP [35] which give information on molecular interaction data and experimentally verified protein-protein interactions.

**Figure 2:**
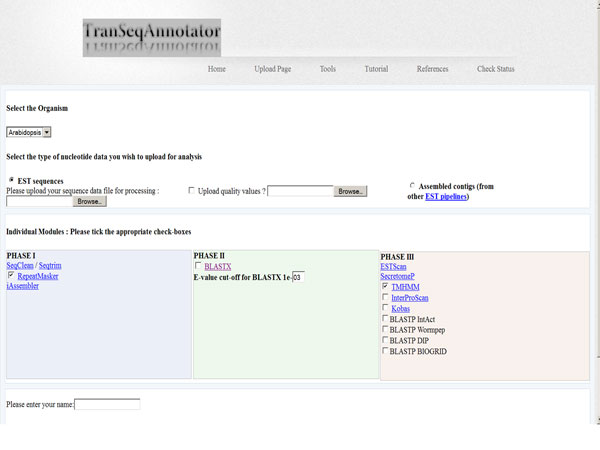
**TranSeqAnnotator data submission page**.

TSA accepts a dataset submitted by the user and optional programs can be selected as required (Figure 2). The progress of the analysis is monitored on the status page which is updated after each selected process is completed and the output of each program is available along with a summarized output. Some of these tools are provided in the ESTExplorer [36] and EST2Secretome [37] pipeline but, the analysis of large-scale EST dataset and short read sequences with updated bioinformatics tools is incorporated with TranSeqAnnotator as part of the benchmarking with the large-scale analysis of *Teladorsagia circumcincta *dataset (unpublished work). Also, the program SecretomeP showed the identification of important proteins which the previous pipelines failed to identify with SignalP. The identification of both classically and non-classically secreted proteins with secretomeP is the highlight of the robust analysis pipeline as our earlier analysis on *Fasciola hepatica *[38].

### Software/hardware environment

TranSeqAnnotator is developed using PERL v5.10.0 which links the different bioinformatics programs and MySQL as backend for data management and analysis. The front end is developed using PHP and the processes are run based on CPU availability. Each input sequence submitted by the user is tagged with a request ID to trace the process. The pipeline runs on a 16-node Linux cluster (2.4 GHz, Intel(R) Xeon (R) CPU, 16 Processors, 32 GB RAM) running on ubuntu server operating system. The output files for viewing and downloading are provided as final results which are available for a week.

## Results and discussion

### Application of TranSeqAnnotator

*Ascaris lumbricoides*, the soil-transmitted helminths or geohelminths is the largest common intestinal nematode parasites of human that causes the disease ascariasis [39]. It infects an estimated 1.2 billion people worldwide, but is usually asymptomatic [40]. 1822 *A. lumbricoides *EST sequences from dbEST [41], were analysed using the TranSeqAnnotator. The dataset is from the adult male whole body *Ascaris lumbricoides *cDNA clone. The phase I of pre-processing (SeqClean and RepeatMasker) aligned/clustered using CAP3 followed by assembly, was carried out which yielded 236 contigs and 658 singletons. These rESTs were mapped to the non-redundant (NR) dataset using BLAST, for nucleotide level annotation. Using a translational matrix, ESTScan conceptually translates these high quality rESTs, which are then transferred to Phase II of TSA, for the prediction of ES proteins, by sequentially running SecretomeP (with a threshold value for the NN-score of 0.9) and TMHMM programs. The cluster dataset, translated peptide sequences and ES proteins were annotated with biochemical pathways, employing KOBAS, domain/family motif and GeneOntology using InterProScan. The query sequences were compared using BLASTP against Wormpep [32] and against the IntAct database (version 1.7.0) to extract all interaction partners. The 894 rESTS were conceptually translated to yield 510 peptide sequences. The GO terms were identified for these putative protein sequences using InterProScan, with 108 peptide sequences assigned biological process (BP), 156 associated with molecular function (MF) and 83 as part of a cellular component (CC) (Additional File 1). The analysis revealed that *translation *(GO:0006412) and *oxidation-reduction process *(GO:0055114) were the highly represented GO categories signifying biological processes. The major number of GO terms in molecular function was *structural constituent of ribosome *(GO:0003735), *oxidoreductase activity *(GO:0016491) and *ATP binding *(GO:0005524) whereas in cellular component, the highly represented GO terms were *ribosome *(GO:0005840) and *extracellular space *(GO:0005615).

A total of 239 peptide sequences were mapped to 113 KEGG pathways using KOBAS. The main KEGG pathways mapped included *ribosomal protein assembly pathway *(n = 34) and *cytoskeleton proteins *(n = 19). Other well represented pathways include *tight junction *(n = 14), *regulation of actin cytoskeleton *(n = 12), *focal adhesion *(n = 12), *valine, leucine and isoleucine degradation *(n = 8) and *propanoate metabolism *(n = 7). Peptides were mapped to several pathways, including *glycolysis/gluconeogenesis, MAPK signaling pathway *and *ubiquitin mediated proteolysis *(Additional File 2).

Domain mapping by Interproscan provides details as to the family, fold and functional domains present in the putative peptides. The most represented was the *collagen triple helix repeat of proteins*, comprising 14 protein entries, followed by *C-type lectin fold *and *transthyretin-like family*, with nine protein entries each. Other highly represented domains are the *actin-like *and *C-type lectin *(Additional File 3).

A total of 32 were predicted by SecretomeP. Of these, 6 are classically secreted peptides; with N-terminal signal sequences while 26 are non-classical, supporting the use of SecretomeP vs. SignalP alone, which can only predict classically secreted proteins. Of these 32, six proteins with transmembrane helices, predicted by TMHMM were eliminated, resulting in 26 excreted/secreted proteins inferred from the present dataset of 894 rESTs. We could identify cecropin (including the cecropin-P1, cecropin-P2, cecropin-P3), cathepsin L from *Ascaris suum *and cathepsin L-like protease from *Strongylus vulgaris*, chymotrypsin/elastase isoinhibitor 1 from *Ascaris suum*, C-type lectin protein 160 from *Ascaris suum *and C-type lectin domain-containing protein 160 from *Ascaris suum*. Gelsolin from *Ascaris suum *and GelSoliN-Like family member (gsnl-1) from *Caenorhabditis elegans *were also identified (Additional File 4). Cecropins, represent a large family of antibacterial and toxic peptides are known to execute host defence functions mainly against micro-organisms [42,43] and are found in insects [44]. Ascaris cecropins (P1-P4) were identified as antimicrobial peptides that were positively inducible by bacterial injection. Ascaris cecropins synthesized chemically were bactericidal against a wide range of microbes, i.e. Gram-positive (*Staphylococcus aureus*, *Bacillus subtilis *and *Micrococcus luteus*) and Gram-negative (*Pseudomonas aeruginosa*, *Salmonella typhimurium*, *Serratia marcescens *and *Esherichia coli*) bacteria, and were weakly but detectably active against yeasts (*Saccharomyces cerevisiae *and *Candida albicans*) [45]. A large family of proteins that binds carbohydrate moieties in a Ca2+-dependent manner are represented by C-type lectins (CTLs) which act as a pathogen recognition molecule or an antibacterial protein in immune responses to protect the worm itself against microbial infection [46-49]. They also play vital role in immune homeostasis by endogenous 'self' ligand recognition [50], and they themselves have a bactericidal activation [51]. Studies have shown that *A. suum *C-type lectin-1(As-CTL-) shows high similarity to *Toxocara canis *C-type lectin (Tc-CTLs) and are exposed to attack by host immune responses. Hence, to avoid protective immune responses in infected animals during tissue migration *A. suum *larvae might interfere with host inflammation processes by As-CTL-1 [52]. The Gelsolin family belongs to a group of actin binding proteins are known to be involved in cell structure, motility, apoptosis, amyloidosis and cancer. Gelsolin-like protein-1 (GSNL-1) from *C. elegans *is a new member of the gelsolin family of actin regulatory proteins which provide new insight into functional diversity and evolution of gelsolin-related proteins [53,54]. We were able to functionally assign GO terms to 26 putative ES proteins with proteolysis (GO:0006508) the most common GO category representing biological processes, cysteine-type peptidase activity (GO:0008234) in molecular function and extracellular region (GO:0005576) in cellular component. Protein processing in endoplasmic reticulum, phagosome, lysosome, antigen processing and presentation, rheumatoid arthritis represented the sequences mapped to KEGG pathways using KOBAS. The TranSeqAnnotaor methodology was benchmarked using the large-scale dataset of *Teladorsagia circumcincta *(unpublished work) and applied for the annotation of *A. lumbricoides*.

### Future directions

TranSeqAnnotator currently supports nucleotide, short reads, protein and ES level annotation. Our aim is to extend the pipeline with updating the masking the repeats with repeatless libraries to annotate newly sequenced organisms and also to carry out annotations for different datasets like RNA-seq, microarray datasets.

## Competing interests

The authors declare that they have no competing interests.

## Authors' contributions

RM carried out the analysis, computational studies and drafted the manuscript. RM, GG, SR and RBG participated in the design of the study and interpretation of data. SR and RBG conceived the project and finalized the manuscript. All authors have read and approved the final manuscript.

## Supplementary Material

Additional file 1 **GO annotation for putative peptides**. Gene Ontology annotations from Interproscan reported. Click here for file

Additional file 2 **KEGG Pathway analysis of proteins (E-value threshold of 1E-05)**. Database matches reported. Click here for file

Additional file 3 **Domain description for the protein sequences**. Interproscan domains reported. Click here for file

Additional file 4 **Top BLAST hits for secreted proteins**. Non-redundant database matches reported. Click here for file
